# What is so complicated about prenatal testing for Down syndrome? A personal view

**DOI:** 10.1007/s00439-021-02292-1

**Published:** 2021-05-17

**Authors:** Louise Bryant

**Affiliations:** School of Medicine, University of Leeds, Worsley Building, Clarendon Way, Leeds, LS2 9JT UK

In a number of ways, I am a personal stakeholder in the debate about prenatal testing for Down syndrome. I am a woman who has benefited from technologies that have enabled me to control my fertility and make my own reproductive choices. I am the sibling of a much-loved person with Down syndrome. It so happens that in addition, I have researched and written about the psychosocial and ethical aspects of prenatal testing for over 20 years. This quote from Barbara Katz Rothman pretty much captures it for me:

“I have never, ever, in my life come across anything as complicated as prenatal testing. Morally, psychologically, politically, socially—on every level, I have never come up against anything as difficult.” (Rothman 1997)

To this list of difficulties, I would add ‘personally’. My brother, born in the 1970s, changed my life and that of my family in so many positive ways despite the negative predictions my parents were subjected to. As a child and teenager, I felt that anyone who knew this truth about having a family member with Down syndrome would not consider testing for or terminating a pregnancy because of the condition. As a woman I became more aware of hard-won reproductive freedoms and even encountered those who like me had a family member with Down syndrome but did not share my views or experience. In my first piece of research with sisters of a sibling with Down syndrome, positive feelings about the sibling were very evident, but one‐third of participants viewed the impact on themselves and their family as less positive, and this was reflected in them holding favourable attitudes towards prenatal testing and termination (Bryant et al. 2005).

It can certainly be argued that many things have changed for the better since that research was conducted, including an increasing awareness that being a parent of a child with Down syndrome is something to celebrate not fear. Still, people with Down syndrome vary in their abilities and physical health, and some have greater educational, support or healthcare needs than others. The rights and lives of people with a learning disability are always precarious; something highlighted once again during the Covid-19 pandemic (Lodge 2020). Many disabled people and their families report struggling ‘against the system’ to access the education, services and employment opportunities they have a right to (Horridge et al. 2019). The public battle for equality, as well as continued negative, and sometimes openly cruel, attitudes towards those with an intellectual disability can constrain the choices women feel they have. It is disingenuous at the very least to assume individual choices are always evidence of autonomy when they may also be evidence of a society’s inability to include and support people with intellectual disability. In my work, hearing the personal experiences of women who choose to terminate a pregnancy for Down syndrome as well as those who choose not to, has led me to fully respect the decisions that *individual* women and their partners make. The reality that screening leads to a reduction in the *population* of people with Down syndrome is more personally difficult and complicated.

Advocacy groups including ‘Do not Screen Us Out’ in the UK argue that government funding of a screening programme for Down syndrome is discriminatory, and that a diagnosis of the condition is not a valid reason to routinely offer termination of pregnancy (Thomas and Rothman 2016). Heidi Crowter, a self-advocate living with Down syndrome, has called for a judicial review to challenge the current English law that allows parents to terminate a pregnancy for Down syndrome up to full-term (BBC 2020). The groups are concerned that the lives of people with Down syndrome and their families are inaccurately and negatively portrayed by health professionals whose own assumptions and values undermine the ability of women to make a fully informed choice (Enoch 2019). They have long raised concerns that the introduction of non-invasive Prenatal Testing (NIPT) into the UK’s National Health Service will lead to a significant reduction in the population of people with Down syndrome.

NIPT, as an earlier and more accurate screening test, is in general viewed favourably by pregnant women some of whom seek access to it through private services. Not all pregnant women choose to screen for Down syndrome (or Edwards’ syndrome or Patau’s syndrome now part of the combined first trimester screening test). Not all women who receive a screen positive result choose diagnostic testing. Not all women who receive a diagnosis of Down syndrome terminate the pregnancy, but around 90% (in most of the UK) do. This proportion has changed remarkably little in the time I have been researching in the area (Mansfield et al. 1999), despite uptake of prenatal screening over this period increasing, and public attitudes towards people with Down syndrome becoming more positive (Henderson and Redshaw 2017). Knowledge of, and attitudes towards Down syndrome, may, however, be less important in a decision to choose screening than some other factors such as a desire for reassurance about foetal health early in pregnancy (Bryant et al. 2010). If the introduction of NIPT into the NHS leads to an increased uptake of screening tests across the pregnant population, then it is likely the number of terminations for Down syndrome will also increase de facto. Early data does support this increase in termination numbers (Van den Bogaert et al. 2021), although termination rates themselves may remain stable (Hill et al. 2017).

Advancing maternal age is associated with an increased chance of having a baby with Down syndrome. In the absence of screening, the number of live births of babies with Down syndrome would almost certainly have grown in line with the rise in average maternal age (de Graaf et al. 2020; Lou et al. 2018); although the knowledge that prenatal screening exists may well play a part in the choices women make about the timing of their pregnancies. Parents have been vocal in demanding support for their children with Down syndrome and instrumental in challenging negative stereotypes. In an imagined world where no prenatal testing existed, more children with Down syndrome would have been born, and many perhaps within families with the resources to be agents of positive social change. I sometimes wonder what life for my brother would be like if there were more, rather than fewer, people with Down syndrome in our society. Personal contact with disabled people is associated with more positive attitudes towards disability (Keith et al. 2015), which in turn are associated with less favourable attitudes to termination of an affected pregnancy (Bryant et al. 2010). Over time, a significant reduction in the number of people with Down syndrome would reduce opportunities for personal interaction and so potentially further impact on personal attitudes. This is not an argument against the rights of individual women to screen for, or have a termination of pregnancy for Down syndrome, but a personal reflection on the complicated nature of prenatal testing as a social as well as a medical technology.

A clear understanding of the impact of NIPT on the global population of people with Down syndrome will not be available for some years and at this point in history, concerns about an imminent ‘world without Down syndrome’ can neither be fully supported nor dismissed. In the meantime, the quality of the lives of people with Down syndrome like my brother, and their family members like me, continue to be subject to public scrutiny and debate. Those, including myself, who support the right for women to make individual prenatal testing and termination decisions must be careful not to justify this right at the expense of the humanity and value of people with Down syndrome.
